# Semen parameters are seriously affected in acephalic spermatozoa syndrome

**DOI:** 10.1186/s12610-022-00170-y

**Published:** 2022-08-26

**Authors:** Li-juan Ying, Lin Yu, Tingting Yang, Ying-bi Wu, Jin-yan Xu, Ye-lin Jia, Yan Zheng, Fuping Li

**Affiliations:** Department of Andrology/Sichuan Human Sperm Bank, West China Second University Hospital, Sichuan University; Key Laboratory of Birth Defects and Related Diseases of Women and Children (Sichuan University), Ministry of Education, No. 1416, Section 1, Chenglong Avenue, Sichuan 610066 Chengdu, China

## Abstract

**Background:**

Previous studies have reported that some patients with headless spermatozoa have poor semen quality, but there has been no published systematic analysis of semen quality in patients with different proportions of headless spermatozoa in semen. We aimed to explore the association of acephalic spermatozoa syndrome and semen quality in men with distinct proportions of headless spermatozoa.

**Material and methods:**

Semen parameter values in patients for whom headless spermatozoa were found in the ejaculates was studied and compared to that of 413 age-matched prenatal examination patients. All semen samples were analyzed following the same methodology in a single laboratory.

**Results:**

All semen parameter values except semen volume were negatively (*P* < 0.05) correlated with the proportion of headless spermatozoa. The semen samples were divided into four groups on the basis of the proportion of headless spermatozoa (PHS) as follows: 0 < PHS ≤ 5% (*n* = 172, Group A1); 5 < PHS ≤ 10% (*n* = 76, Group A2); 10 < PHS ≤ 20% (*n* = 71, Group B); and PHS > 20% (*n* = 71, Group C). In Group A1, only one semen parameter value (progressive motility) was lower than those of the control group, but in Group A2, this increased to five (sperm vitality, normal sperm morphology, sperm motility, VCL (curvilinear velocity) and ALH (amplitude of lateral head displacement)). Worse still, all semen parameter values were significantly lower in Group B and Group C than in the control group (*P* < 0.05).

**Conclusions:**

Semen samples containing headless spermatozoa tend to have lower quality than samples without headless spermatozoa. Increases in the proportion of headless spermatozoa in semen are associated with decreased semen quality. We suggest that headless spermatozoa should be seriously assessed and accurately counted in semen analysis, especially for ejaculate in which the proportion of headless spermatozoa exceeds 5%.

## Résumé

**Contexte:**

Des études antérieures ont rapporté que certains patients qui avaient des spermatozoïdes sans tête présentaient une mauvaise qualité du sperme, mais il n’y a eu aucune analyse systématique publiée sur la qualité du sperme chez les patients ayant des proportions différentes de spermatozoïdes sans tête dans leur sperme. Nous avons cherché à explorer l’association entre syndrome des spermatozoïdes acéphaliques et qualité du sperme chez les hommes ayant des proportions distinctes de spermatozoïdes sans tête. Les valeurs des paramètres du sperme chez les patients pour lesquels des spermatozoïdes sans tête ont été trouvés dans l’éjaculat ont été étudiées et comparées à celles de 413 patients consultant pour un examen prénatal, appariés sur l’âge. Tous les échantillons de sperme ont été analysés selon la même méthodologie dans un seul laboratoire.

**Résultats:**

Toutes les valeurs des paramètres du sperme, à l’exception du volume de sperme, étaient négativement (*P* < 0,05) corrélées avec la proportion de spermatozoïdes sans tête. Les échantillons de sperme ont été divisés en quatre groupes sur la base de la proportion de spermatozoïdes sans tête (PHS) comme suit: 0 < PHS ≤ 5% (*n* = 172, groupe A1); 5 < PHS ≤ 10% (*n* = 76, groupe A2); 10 < PHS ≤ 20% (*n* = 71, groupe B); et PHS > 20% (*n* = 71, groupe C). Dans le groupe A1, une seule valeur de paramètre de sperme (motilité progressive) est. inférieure à celle du groupe témoin, mais dans le groupe A2, le nombre s’élève à cinq (vitalité des spermatozoïdes, morphologie normale des spermatozoïdes, mobilité des spermatozoïdes, VCL (vitesse linéaire curviligne) et ALH (amplitude du déplacement latéral de la tête)). Pire encore, toutes les valeurs des paramètres du sperme étaient significativement plus faibles dans les groupes B et C que dans le groupe témoin (*P* < 0,05).

**Conclusions:**

Les échantillons de sperme contenant des spermatozoïdes sans tête ont tendance à avoir une qualité inférieure à celle des échantillons dépourvus de spermatozoïdes sans tête. L’augmentation de la proportion de spermatozoïdes sans tête dans le sperme est. associée à une réduction de la qualité du sperme. Nous suggérons que les spermatozoïdes sans tête devraient être sérieusement évalués et comptés avec précision dans l’analyse du sperme, en particulier pour l’éjaculat dans lequel la proportion de spermatozoïdes sans tête dépasse 5%.

## Introduction

Reproductive health is essential for ensuring the continuity of human populations. However, recent reports indicate that approximately 15% of couples suffer from fertility problems, and it is known that up to half of cases of infertility may be due to male factors [1, 2]. Teratozoospermia is an important cause of male infertility. Headless spermatozoa are severely deformed cells that have only a flagellum. This abnormal morphology was first described as “minute-head sperm”, but in 1981, Perotti et al. demonstrated that minute-head sperm actually have no head at all and that the “minute heads” were actually small cytoplasmic droplets [3]. Headless sperm are produced when there are abnormalities in the formation of the sperm head-tail coupling apparatus (HTCA), an important structure that anchors the sperm flagellum to the sperm head [4, 5].

Acephalic spermatozoa syndrome has been confirmed to cause male infertility, as the semen contains many headless spermatozoa [4, 6–9]. Kamal reported few headless spermatozoa in semen, suggesting that spermatozoa are easily decapitated, which may also be the cause of male infertility or failure of assisted reproduction [10]. Moreover, both the incidence of headless spermatozoa and their proportion are higher in the infertile population than the fertile; the percentage of headless spermatozoa in fertile men is 2.7 ± 3.1%, while in infertile men, it is 9.0 ± 8.8% [11, 12]. Therefore, the proportion of headless spermatozoa might be a cause of infertility or impaired fertility in males.

Previous studies have reported that some patients with headless spermatozoa in ejaculates have poor semen quality [13, 14], but until now, there has been no systematic analysis of semen quality in patients with different proportions of headless spermatozoa. In this study, we aimed to explore the effect of acephalic spermatozoa syndrome on semen quality by evaluating the semen parameter values in samples containing distinct proportions of headless spermatozoa.

## Materials and methods

### Study population

**Fig. 1:**
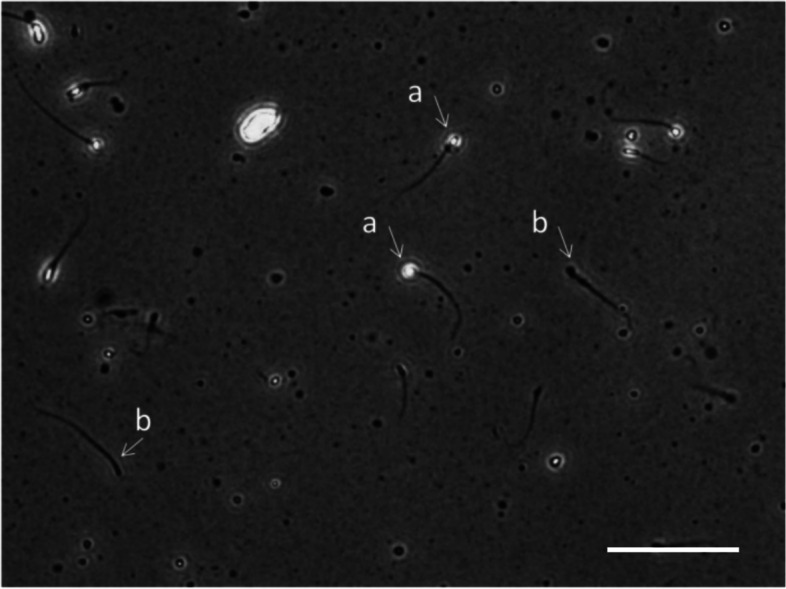
Photomicrograph of wet preparation of the ejaculated. Headless spermatozoa (**a**) and intact spermatozoa (**b**) are observed. Scale bar: 50 μm

Between January 2018 and July 2019, we selected 391 patients for whom headless spermatozoa (defined as having a flagellum only and no head; see Fig. 1) were found in the ejaculates as study and 413 age-matched prenatal examination patients as control among the andrology reproductive clinic outpatients of West China Second University Hospital, Sichuan University. All patients were subjected to routine semen analysis after an abstinence period of 2–7 days at our andrology laboratory with the same methodology. Men with azoospermia, an abstinence period beyond 2–7 days or incomplete semen collection were excluded. In the control group, headless spermatozoa were found in the semen samples of 13 participants，and the proportion of the headless spermatozoa was 11.6 ± 16.1%(mean ± SD). Quality assurance monitoring was performed according to WHO guidelines from 2010 [15]; the quality assurance program comprises monthly monitoring of semen analysis results to identify systematic errors. Two-way analysis of variance was performed to compare the results of analyses of the same semen samples by all technicians of our laboratory to assess systematic differences among technicians; sperm concentration quality control sample analysis was included in the laboratory as part of the regular workload and to monitor the results using quality control charts to identify random and systematic errors in concentration analysis. During the study period, no errors in quality control were discovered. In addition, the body mass index (BMI) was calculated via weight in kg and height in musing related formula. Through a face-to-face interview, demographic information including age, smoking, reason for visit (prenatal examination or infertility) was collected.

### Semen analysis

Semen samples were obtained by masturbation, and after liquefaction in a 37 °C incubator, the volume, sperm vitality and sperm morphology of all samples were analyzed according to WHO guidelines (5th editions) [15]. The volume of the samples was measured by weighing the collection container. After staining with the eosin Y technique, we calculated the sperm vitality (percentage of live heads-intact cells). We analyzed intact cell morphology (percentage of normal sperm morphology) after Papanicolaou staining according to the standards in the WHO manual (5th editions). The sperm concentration and sperm motility (percentage of progressive motility) were evaluated in a Makler counting chamber with the help of computer-assisted sperm analysis (CASA) (SSA-II, Suijia, China) and checked manually, which also provides the following objective sperm motility parameters: straight-line velocity (VSL), curvilinear velocity (VCL), average path velocity (VAP), beat cross frequency (BCF), and amplitude of lateral head displacement (ALH). Every sample was analyzed at least twice and evaluated at least 200 spermatozoa in at least five fields. Through our verification, this concentration analysis method is comparable with the method recommended by the WHO manual (fifth edition). Moreover, it is worth noting that only intact spermatozoa (defined as having both the head and tail; see Fig. 1) were assessed in the analysis of the sperm concentration, sperm vitality, sperm morphology, sperm motility and sperm motility parameters. Headless spermatozoa were not counted. The headless spermatozoa concentration and round cell concentration were obtained under an optical microscope through a manual method in the Makler counting chamber, and the concentration was estimated in relation to all spermatozoa, for instance, “25 headless spermatozoa per 100 spermatozoa”. The percentage of headless spermatozoa was calculated as the headless spermatozoa concentration/ (headless spermatozoa concentration + whole spermatozoa concentration) × 100%.

### Statistical analysis

The results from the different groups were compared by the nonparametric Mann–Whitney test for continuous data and the chi-square test for categorical variables. Each group of statistics is indicated with medians (interquartile ranges) or frequencies (proportions). Spearman correlation coefficients were determined for different proportions of headless spermatozoa and semen parameters. The distribution curve was applied to explore the distribution of the proportion of headless Spermatozoa. We also constructed receiver operating characteristic (ROC) curves to assess the specific proportion of headless spermatozoa, may be predictive of abnormal semen parameters. All evaluations were performed using PRISM software. Differences between groups were considered statistically significant at *P* < 0.05.

## Results

### Characteristics of the headless spermatozoa and control groups

Compared with the control group, the median intact sperm concentration, total sperm count, sperm vitality, progressive motility, normal sperm morphology and some sperm motility parameters (VCL, ALH, VAP, BCF) were lower in the headless spermatozoa group (*p* < 0.05). However, there were no significant differences between the headless spermatozoa group and the control group in semen volume, number of round cells, or VSL of intact cells (*p* > 0.05) (Table 1). Moreover, a notable morphological difference was observed between the two groups; the proportion of spermatozoa with a small acrosomal area (less than 40% of the sperm head is occupied by the acrosome) or with no acrosomal area in the ejaculate was observed in 27.6% (108/391) of the patients in the headless spermatozoa group and 3.4% (14/413) of the patients in the control group, and the percentage more than 20%.

**Table 1:** General characteristics of the headless spermatozoa group and control group

	Headless spermatozoa group	Control group	*P*-value
N	391	413	
Demographic information			
Age (year)	30(27–32)	29(27–34)	0.9343
BMI (kg/m2)	23.9 (22.7–26.5)	24.5 (23.2–26.7)	0.1363
Prenatal examination patients (no. [%])	42(2.5%)	413(100%)	< 0.0001
PHS(%)	0(0–0)	38(26–56)	< 0.0001
Current smoker (no. [%])	42(10.7%)	51(12.3%)	0.4764
Abstinence delay (day)	4(3–5)	3(3–5)	0.4842
Semen parameters			
Semen volume (ml)	3.3(2.5–4.1)	3.4(2.6–4.4)	0.9654
Round cells (millions /ml)	0.2(0.2–0.5)	0.3(0.2–0.5)	0.0610
Sperm concentration (millions/ml)	73.8(36.9–131.6)	87.3(53.3–138.6)	0.0034
Total sperm count (millions/ejaculate)	236.5(108.7–419.4)	296.6(178.1–439.5)	0.0005
Sperm vitality (%)	77(70–81)	79(72–84)	0.0001
Progressive motility (%)	49(37–61)	59(45–68)	< 0.0001
Normal sperm morphology (%)	3.9(2–6)	4.9(3–6.9)	< 0.0001
VCL (μm/sec)	35.1(25.0–45.2)	38.2(28.5–47.3)	0.0109
VAP (μm/sec)	25.3(17.6–32.7)	27.6(20.4–33.9)	0.0245
ALH (μm/sec)	3.14(2.3–3.9)	3.3(2.5–4.2)	0.0025
BCF (Hz)	9.9(7.6–12.1)	10.6(8.5–12.5)	0.0092

*BMI* Body mass index, *PHS* Headless spermatozoa proportion, *VSL* Straight-line velocity, *VCL* Curvilinear velocity, *VAP* Average path velocity, *BCF* Beat cross frequency, *ALH* Amplitude of lateral head displacement. *n* = the number of samples. Data are presented as the Median (25th centile, 75th centile), and comparisons between the headless sperm group and the control group were determined by the nonparametric Mann-Whitney test for continuous data and the chi-squared test for categorical variables

### Semen parameters and proportion of headless spermatozoa in semen

To explore the relationship between the proportion of headless spermatozoa and semen quality, we correlated the proportion of headless cells with the parameter values of head-intact cells in the headless sperm group. All the semen parameters except semen volume were negatively (*P* < 0.05) correlated with the proportion of headless spermatozoa (Table 2).

**Table 2:** Correlation analyses between sperm parameters and headless sperm proportion

	Median (25th centile,75th centile)	Correlation coefficient	P
Age (year)	30(27–32)	− 0.09212	0.0678
Abstinence delay (day)	4(3–5)	− 0.03669	0.4700
Semen volume (ml)	3.3(2.5–4.1)	0.06314	0.2134
Sperm concentration (millions /ml)	73.8(36.9–131.6)	−0.4308	< 0.0001
Total sperm count (millions/ejaculate)	236.5(108.7–419.4)	−0.4618	< 0.0001
Sperm vitality (%)	77(70–81)	−0.1718	0.0007
Progressive motility(%)	49(37–61)	−0.2740	< 0.0001
Normal sperm morphology(%)	8(2–6)	−0.1752	0.0006
VCL (μm/sec)	35.1(25.0–45.2)	−0.3038	< 0.0001
VAP (μm/sec)	25.3(17.6–32.7)	−0.2991	< 0.0001
ALH (μm/sec)	3.14(2.3–3.9)	−0.3517	< 0.0001
BCF (Hz)	9.9(7.6–12.1)	−0.3718	< 0.0001

Correlation results between sperm parameters and headless sperm proportion are expressed by the Spearman correlation coefficient and its corresponding *p* value. *VSL* Straight-line velocity, *VCL* Curvilinear velocity, *VAP* Average path velocity, *BCF* Beat cross frequency, *ALH* Amplitude of lateral head displacement

**Fig. 2:**
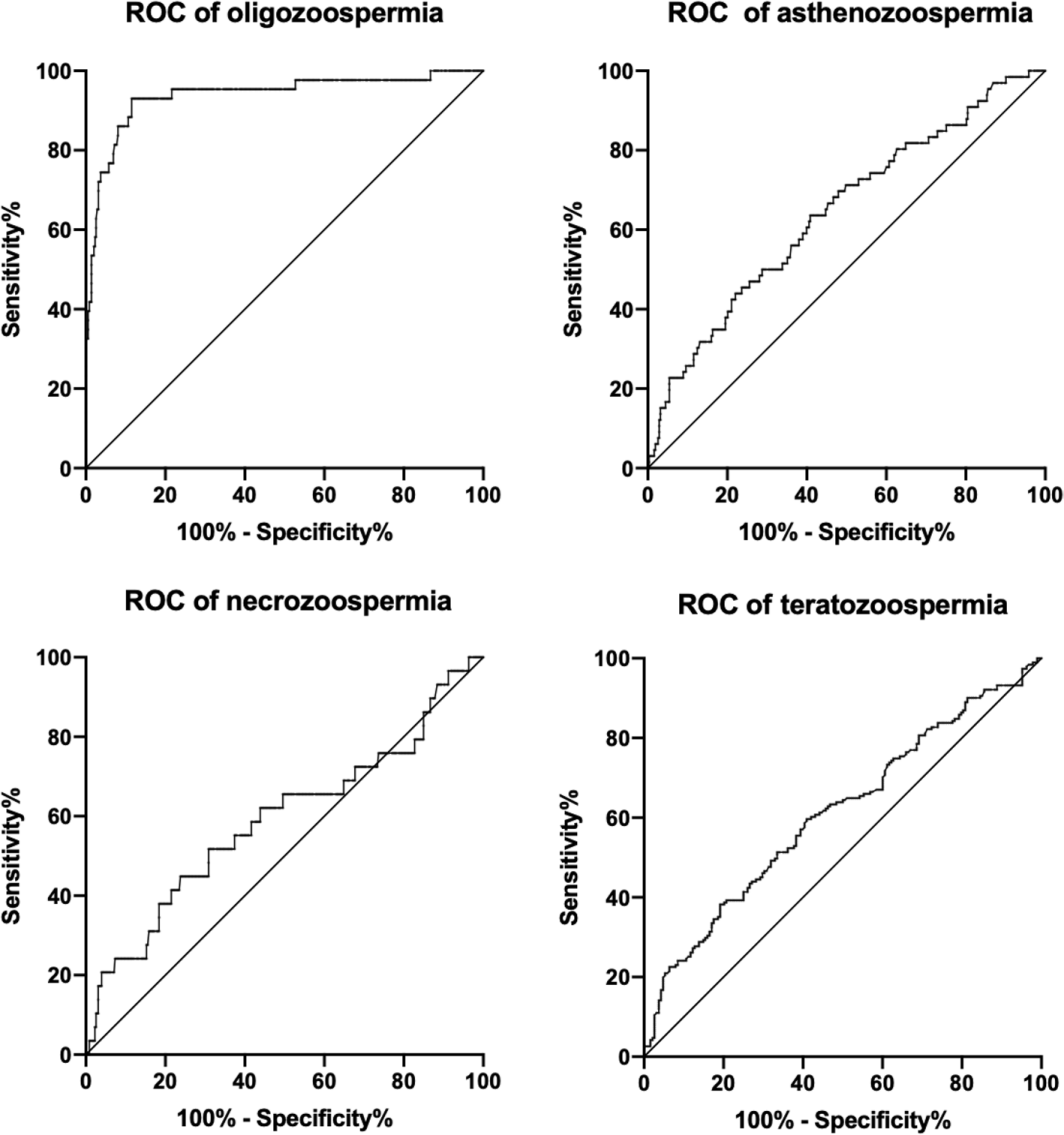
Receiver operating curve. Receiver operating characteristic (ROC) curves to assess the specific proportion of headless spermatozoa that may be predictive of the following: oligozoospermia (intact sperm concentration < 15*106 spermatozoa/mL), asthenozoospermia (the percentage of intact normal morphology sperm< 4%), necrozoospermia (the percentage of intact live sperm < 58%) and teratozoospermia (the percentage of intact normal morphology sperm< 4%)

**Fig. 3:**
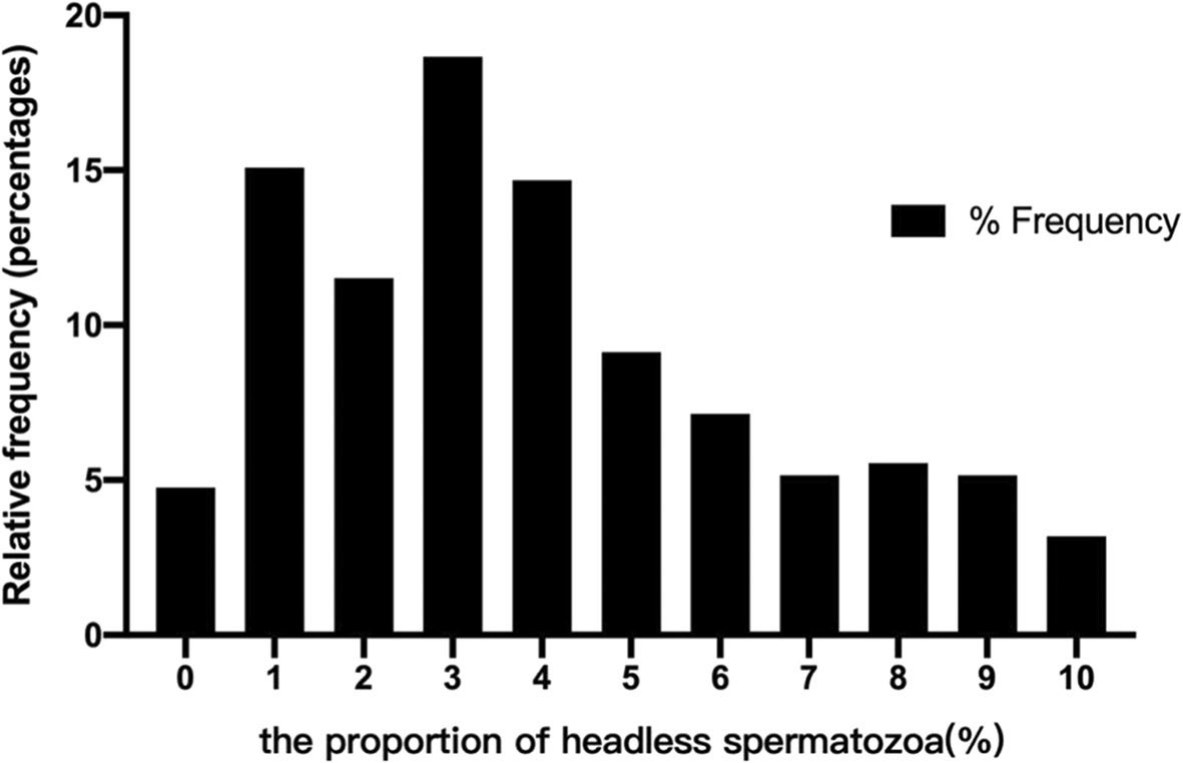
Distribution curve. The distribution of the proportion of headless spermatozoa (PHS) in group A (0 < PHS ≤ 10%)

**Fig. 4:**
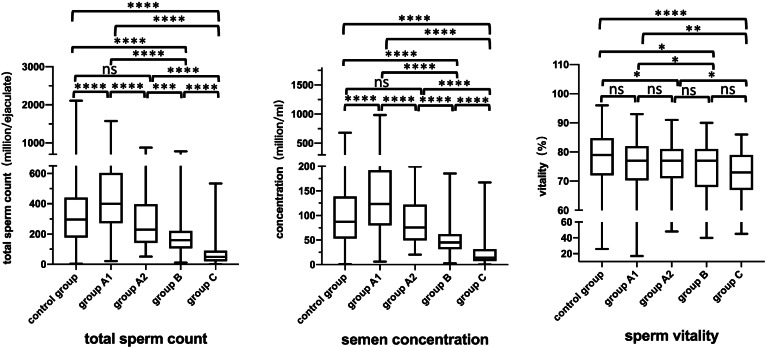
The proportion of headless spermatozoa and semen parameter values (intact cells). Box plot displaying the minimum, 25th, median, 75th, and maximum values and of total sperm count, sperm concentration and sperm vitality according to the various groups of the proportion of headless spermatozoa. Group A1 = 0 < PHS ≤ 5%, group A2 = 5 < PHS ≤ 10%, group B = 10 < PHS ≤ 20%, group C=PHS > 20%. Differences between groups were investigated by the nonparametric Mann-Whitney test. * *P* < .05; ** *P* < .005; *** *P* < .0005; **** *P* < .0001

**Fig. 5:**
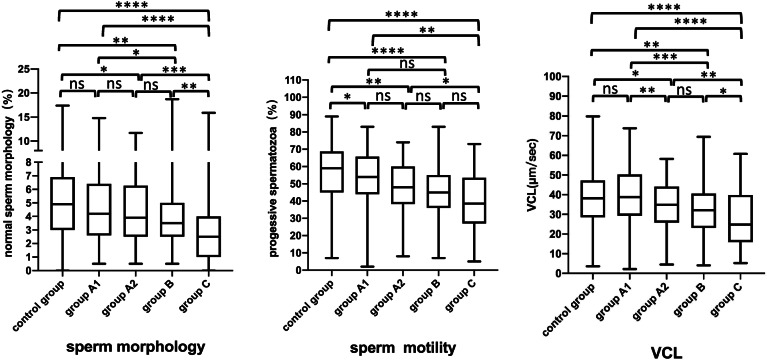
The proportion of headless spermatozoa and semen parameter values (intact cells). Box plot displaying the minimum, 25th, median, 75th, and maximum values and of normal sperm morphology, progressive sperm motility and VCL (curvilinear velocity) according to the various groups of the proportion of headless spermatozoa (PHS). group A1 = 0 < PHS ≤ 5%, group A2 = 5 < PHS ≤ 10%, group B = 10 < PHS ≤ 20%, group C=PHS > 20%. Differences between groups were investigated by the nonparametric Mann-Whitney test. * *P* < .05; ** *P* < .005; *** *P* < .0005; **** *P* < .0001

**Fig. 6:**
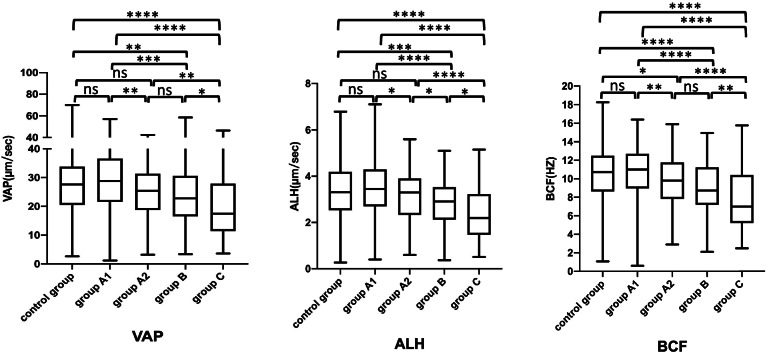
The proportion of headless spermatozoa and semen parameter values (intact cells). Box plot displaying the minimum, 25th, median, 75th, and maximum values and of VAP (average path velocity), ALH (amplitude of lateral head displacement) and BCF (beat cross frequency) according to the various groups of the proportion of headless spermatozoa. Differences between groups were investigated by the nonparametric Mann-Whitney test. * *P* < .05; ** *P* < .005; *** *P* < .0005; **** *P* < .0001

To identify the appropriate cut-off value for the specific proportion of headless spermatozoa that may be predictive of abnormal semen parameter values, we selected the WHO (2010) sperm concentration (15*10^6^/ml), sperm motility (32%), and sperm morphology (4%) reference values (lower reference limits) as the cutoff values to draw ROC curves. The headless sperm proportion threshold with the best balance of sensitivity and specificity was calculated for each parameter (sperm concentration, motility, morphology) separately for a better distinction between normal and abnormal values. The cutoff values for proportion of headless sperm were found to be from 7.2% and 18.0% (Table 3, Fig. 2). From these findings, the headless spermatozoa group was divided into three subgroups based on the percentage of headless spermatozoa (PHS) as follows: 0 < PHS ≤ 10% (*n* = 249, group A), 10 < PHS ≤ 20% (*n* = 71, group B) and PHS > 20% (*n* = 71, group C). Compared with the control group, in Groups A, B and C, a decrease was found in the semen parameter values. In Group A, the rates of normal sperm morphology, vitality and motility were lower than those in the control group. Moreover, nearly all the semen parameter values of Group B and Group C were significantly lower than those of the control group (*P* < 0.05) (Table 4). According to the above results, we used a distribution curve to analyze the distribution of the proportion of headless spermatozoa in Group A and found that the proportion of headless sperm was mainly concentrated on the left (Fig. 3); therefore, we tried to further divide Group A into Group A1 (0 < PHS ≤ 5%) and Group A2 (5 < PHS ≤ 10%). Compared with the control group, in Group A1, only one semen parameter value (progressive motility) was decreased. However, in Group A2, there were five semen parameters (sperm vitality, sperm morphology, sperm motility, VCL, ALH) with lower values than those in the control group. In addition, we also observed the trend that the semen parameter values decreased with increasing proportions of headless spermatozoa (Table 5, Figs. 4, 5 and 6).

**Table 3:** Receiver operating curves of headless sperm proportion predicting oligozoospermia, asthenozoospermia, necrozoospermia, and teratozoospermia

	Cut-off	Area	Sensitivity (%)	Specificity (%)
Oligozoospermia	17.96	0.9382	93.02	88.47
Asthenozoospermia	7.15	0.6432	63.64	59.11
Necrozoospermia	13.63	0.5891	44.83	76.2
Teratozoospermia	12.06	0.6109	38.22	80.85

*Note*: Oligozoospermia (intact sperm concentration < 15*106 spermatozoa/mL), Asthenozoospermia (the percentage of intact normal morphology sperm< 4%), Necrozoospermia (the percentage of intact live sperm < 58%), Teratozoospermia (the percentage of intact normal morphology sperm< 4%)

**Table 4:** The comparison of semen parameter values (intact cells) in group A, group B, group C and the control

Groups	Control group	Group A	Group B	Group C
n	413	249	71	71
Sperm concentration (millions /ml)	87.3(53.3–138.6)	108.9(68.3–168.6) ^a^	45.1(31.8–61.1) ^ab^	13.9(7.1–30.8) ^abc^
Total sperm count (millions/ejaculate)	296.6(178.1–439.5)	368.7(200–525.6) ^a^	159.5(106.7–220.7) ^ab^	49.7(20.3–90.5) ^abc^
Sperm vitality (%)	79(72.0–84.0)	77(71.0–82.0) ^a^	77(68.0–81.0) ^a^	73(67.0–79.0) ^ab^
Normal sperm morphology (%)	4.9(3.0–6.9)	4(2.5–6.4) ^a^	3.5(2.5–5.0) ^a^	2.5(1.0–4.0) ^abc^
Progressive motility (%)	59(45–68)	53(40.8–62.3) ^a^	45(36.5–55.0) ^ab^	38.5(27.0–52.5) ^ab^
VCL (μm/sec)	38.2(28.5–47.3)	37.5(28.3–47.1)	32.2(23.2–40.4) ^ab^	24.8(15.9–38.8) ^abc^
VAP (μm/sec)	27.6(20.4–33.9)	27.2(20.4–34.7)	22.8(16.5–30.2) ^ab^	17.5(11.5–26.8) ^abc^
ALH (μm/sec)	3.3(2.5–4.2)	3.4(2.6–4.2)	2.9(2.1–3.5) ^ab^	2.2(1.49–3.1) ^abc^
BCF (Hz)	10.6(8.5–12.5)	10.6(8.5–12.5)	8.7(7.3–11.2) ^ab^	7.0(5.3–10.4) ^abc^

*n* = the number of samples. Data are presented as the median (25th percentile, 75th percentile). group A: 0 < PHS ≤ 10%, group B: 10 < PHS ≤ 20%, group C: PHS > 20%. Comparisons among group A, group B, group C and the control group were determined by the nonparametric Mann–Whitney test. ^a^ indicates a significant difference compared with the control group. ^b^ indicates a significant difference compared with Group A. ^c^ indicates a significant difference compared with Group B

**Table 5:** The comparison of semen parameter values (intact cells) in group A1, group A2, group B, group C and the control

Groups	control group	group A1	group A2	group B	group C
**n**	**413**	**172**	**76**	**71**	**71**
Sperm concentration (millions /ml)	87.3(53.3–138.6)	123.2(80.35–191.075) ^a^	75.7(52.6–119.1) ^b^	45.1(31.8–61.1) ^abc^	13.9(7.1–30.8) ^abcd^
Total sperm count (millions/ejaculate)	296.6(178.1–439.5)	399.5(277.275–598.45) ^a^	229.7(151.4–396.2) ^b^	159.5(106.7–220.7) ^abc^	49.7(20.3–90.5) ^abcd^
Sperm vitality (%)	79(72.0–84.0)	77(70.75–82)	77(71–81) ^a^	77(68.0–81.0) ^a^	73(67.0–79.0) ^abc^
Normal sperm morphology (%)	4.9(3.0–6.9)	4.2(2.8–6.4)	3.9(2.5–6) ^a^	3.5(2.5–5.0) ^ab^	2.5(1.0–4.0) ^abcd^
Progressive motility (%)	59(45–68)	54(44–65.3) ^a^	48(39.5–60) ^ab^	45(36.5–55.0) ^ab^	38.5(27.0–52.5) ^abc^
VCL (μm/sec)	38.2(28.5–47.3)	38.8(29.4–50.0)	34.9(26.3–44.1) ^ab^	32.2(23.2–40.4) ^ab^	24.8(15.9–38.8) ^abcd^
VAP (μm/sec)	27.6(20.4–33.9)	28.8(21.6–36.5)	25.4 (19.0–31.3) ^b^	22.8(16.5–30.2) ^ab^	17.5(11.5–26.8) ^abcd^
ALH (μm/sec)	10.6(8.5–12.5)	11.0(9.0–12.7)	9.8 (7.8–11.8) ^ab^	8.7(7.3–11.2) ^abc^	7.0(5.3–10.4) ^abcd^
BCF (Hz)	3.3(2.5–4.2)	3.5(2.7–4.3)	3.3 (2.4–3.9) ^b^	2.9(2.1–3.5) ^ab^	2.2(1.49–3.1) ^abcd^

*n* = the number of samples. Data are presented as the median (25th percentile, 75th percentile). group A1: 0 < PHS ≤ 5%, group A2: 5 < PHS ≤ 10%, group B: 10 < PHS ≤ 20%, group C: PHS > 20%. Comparisons among group A1, group A2, group B, group C and the control group were determined by the nonparametric Mann–Whitney test. ^a^ indicates a significant difference compared with the control group. ^b^ indicates a significant difference compared with group A1. ^c^ indicates a significant difference compared with Group A2. ^d^ indicates a significant difference compared with Group B

## Discussion

Headless spermatozoa are a specific type of structural defect. Previous research has indicated that patients with a high proportion of headless spermatozoa may face infertility and that their semen quality is usually low [13, 16, 17]. In addition, the incidence and proportion of headless spermatozoa in the infertile population are higher than those in the fertile. For fertile men, the headless spermatozoa proportion is typically below 13% [12]. Our data show that semen parameter values are negatively correlated with the proportion of headless spermatozoa. There was no noticeable reduction in semen quality when the proportion of headless spermatozoa was under 5%, and the semen parameter values declined significantly when the proportion of headless spermatozoa was more than 5%. Therefore, we conclude that a proportion of headless spermatozoa exceeding 5% is accompanied by a reduction in semen parameter values and should be considered in clinical diagnosis.

A large amount of literature has confirmed that headless spermatozoa can be caused by mutations in *SUN5*, *PMFBP1*, *TSGA10*, or *BRDT* [9, 14, 18–22]. Male exposure to khat or methyl chloride, or ligation of the vas deferens, can also lead to the production of headless spermatozoa [23–25]. Investigations in male mice have found that loss-of-function mutations in genes involved in the production of headless spermatozoa, such as *Spata6*, *Hook1*, *Prss21*, *Oaz3*, and *Odf1,* can cause fertility reduction or infertility [26–29]. Interestingly, no mutations of these genes have been identified in humans, which might be explained by either genetic heterogeneity underlying this syndrome or differences in the functions of these genes or in the molecular pathogenesis between mice and humans. Research on the causes of headless spermatozoa has been focused on patients with a high proportion of headless spermatozoa in semen. However, it is very important to also study the causes of lower proportions of headless spermatozoa, which may explain some cases of idiopathic male infertility.

Alterations in any of the above factors could lead to abnormalities in the HTCA structure, in turn causing the sperm neck to be unstable, the detachment of the sperm tail from the head during spermatid elongation, the sperm heads are usually phagocytosed by Sertoli cells, and the flagella release into semen. Alternatively, spermatozoa may fracture when subjected to mechanical stress (mixing, centrifugation or micromanipulation) in vitro, and present in semen as free heads and free flagellums [4, 8, 10, 14, 19, 30, 31]. Sperm concentration as evaluated according to the WHO manual (fifth edition) standards considers only whole spermatozoa (i.e., cells with both a head and a tail), while free tails and heads are not counted. This would explain why a higher proportion of headless spermatozoa is associated with a lower sperm concentration.

Sperm mitochondria play a major role in sperm motility, as they generate adenosine-triphosphate (ATP) [15, 32]. The sperm tail is an important structure for sperm motility, and human sperm swim forward by moving their tail symmetrically from side to side [33]. In transmission electron micrographs, the intact spermatozoa of patients whose semen also contains headless cells often have abnormal structures, such as disassembled mitochondria and sperm tail malformations [16, 34–36]. These morphological abnormalities may be responsible for the decrease in sperm motility. In addition, our data also show that the motility parameters (VCL, ALH, VAP, BCF) of intact spermatozoa are lower in semen samples containing headless spermatozoa, confirming that among intact cells, not only the percentage of progressive motility but also the movement type is altered. These motility parameters were reported to predict the success of intrauterine insemination (IUI) and in-vitro fertilization (IVF) in couples receiving infertility treatment [37–39]. The results indicate that even with the same sperm concentration, motility and normal morphology, males with headless spermatozoa may demonstrate lower fertility.

Our data show that semen samples containing headless spermatozoa present other defects in sperm morphology and a higher incidence of abnormal acrosomes. Previous studies also reported acrosomal abnormalities in intact spermatozoa of semen samples containing headless cells [7, 10, 40]. The acrosome is formed by the trans-Golgi, and it is the unique structure of mature spermatozoa [41]. In 1984, Bacetti et al. reported that headless spermatozoa can occur owing to overproduction of vesicles by the Golgi complex in the region between the centrioles and nucleus [42]. GOPC and VPS54 are important constituent proteins in the Golgi apparatus in tissue culture cells, and knockout mice of the VPS54 or GOPC genes presented abnormal acrosome formation [43–46] Li L et al. confirmed that the expression of Golgi-related genes was upregulated, including GOPC and VPS54, in a patient with acephalic spermatozoa syndrome, suggesting a relationship in the pathology between acephalic spermatozoa and abnormal acrosome formation [20]; our data support their inference. Sperm acrosomes play an important role in spermatozoa binding to the zona pellucida during fertilization, and abnormal acrosomes may be a reason for male infertility or fertility reduction in semen containing headless spermatozoa, reminding us to give more attention to the acrosome function of intact spermatozoa in ejaculations that contain headless spermatozoa.

## Conclusion

Semen samples containing headless spermatozoa tend to have lower semen parameter values than samples without headless spermatozoa. This may be because different proportions of headless sperm are produced by different pathogenic mechanisms, which requires further study. Previous research has reported that patients whose semen contains headless sperm may be infertile or suffer decreased fertility [4, 10, 12, 13, 26–29, 47–49]. Our research supports some of these statements regarding sperm quality. However, the decrease in semen parameter values is only a symptom, and the specific reasons for the decrease need to be further explored in combination with the factors that lead to the production of headless spermatozoa. More importantly, headless spermatozoa should be assessed seriously and counted accurately in semen analysis.

## Data Availability

The datasets generated and analyzed during the current study are not publicly available because local institutional patient data are considered confidential but are available from the corresponding author upon reasonable request.

## Abbreviations

ALH: Amplitude of lateral head displacement
ATP: Adenosine-triphosphate
BCF: Beat cross frequency
BMI: Body mass index
CASA: Computer-assisted sperm analysis
HTCA: Sperm head-tail coupling apparatus
IUI: Intrauterine insemination
IVF: In-vitro fertilization
PHS: Proportion of headless spermatozoa
VAP: Average path velocity
VCL: Curvilinear velocity
VSL: Straight-line velocity
